# A bibliometric analysis of photobiomodulation therapy for the prevention and treatment of chemotherapy- and radiotherapy-induced oral mucositis

**DOI:** 10.3389/froh.2026.1811389

**Published:** 2026-06-09

**Authors:** Madina Kurmanalina, Amin Tamadon, Elnara Ismagulova, Gulnar Sultanova, Aigul Sumanova, Nurzhanat Khaidarova, Aruzhan Aitmukhanbetova

**Affiliations:** 1 School of Dentistry, West Kazakhstan Marat Ospanov Medical University, Aktobe, Kazakhstan; 2 Department of Natural Sciences, West Kazakhstan Marat Ospanov Medical University, Aktobe, Kazakhstan; 3 Departments of Otolaryngology and Ophthalmology, West Kazakhstan Marat Ospanov Medical University, Aktobe, Kazakhstan; 4 Department of Therapeutic and Surgical Dentistry, Astana Medical University, Astana, Kazakhstan; 5 Department of Dentistry and Maxillofacial Surgery, Semey Medical University, Semey, Kazakhstan

**Keywords:** bibliometrics, chemotherapy, oral mucositis, photobiomodulation, radiotherapy

## Abstract

**Introduction:**

Photobiomodulation therapy has emerged as an effective non-invasive intervention for preventing and managing chemotherapy- and radiotherapy-induced oral mucositis, a frequent complication that negatively affects oral health and quality of life in cancer patients.

**Methods:**

This bibliometric study was guided by PRISMA 2020 principles for study identification and selection. A total of 152 publications indexed in Scopus, Web of Science, and PubMed between 2016 and 2025 were analyzed using bibliometric indicators, including publication trends, authorship, institutional productivity, country contributions, citation analysis, and keyword evolution.

**Results:**

The results demonstrated a substantial increase in scientific production between 2016 and 2025, with Brazil, the United States, and France leading research output. The most productive institutions included Universidade Federal de Goiás, Universidade de São Paulo, and Harvard University, while Supportive Care in Cancer and Lasers in Medical Science were the most influential journals. International collaboration networks showed strong global partnerships, and keyword analysis revealed increasing focus on photobiomodulation therapy, oral mucositis, chemotherapy, radiotherapy, and prevention.

**Conclusion:**

These findings indicate rapid global expansion and increasing scientific and clinical relevance of photobiomodulation therapy research, providing valuable insights into scientific development, key contributors, and emerging research directions that support future advances in evidence-based oral healthcare. However, the bibliometric nature of the study, heterogeneity of the included literature, and the lack of direct assessment of clinical outcomes should be considered as limitations.

## 1 Introduction

Oral mucositis (OM) is a frequent and clinically significant complication of cancer therapy that manifests as painful inflammation and ulceration of the oral mucosa, interfering with basic functions such as eating and speaking. In oncologic settings, OM contributes substantially to treatment-related morbidity and negatively impacts patient quality of life and healthcare resource utilization (1–3). Importantly, OM is associated not only with local symptoms but also with systemic complications, including increased risk of infection and treatment interruptions, which may compromise oncologic outcomes (4). Recent clinical evidence continues to highlight the high incidence of OM in patients receiving cytotoxic therapies, particularly in head and neck oncology and high-dose chemotherapy contexts (5–7).

Photobiomodulation therapy (PBMT)—formerly described as low-level laser therapy (LLLT)—employs non-ionizing red and near-infrared light to modulate cellular activity and enhance tissue repair processes (8). The proposed mechanisms of PBMT include reducing inflammation, improving microcirculation, and stimulating mitochondrial metabolism, which may attenuate the pathophysiologic progression of OM (9, 10). At the cellular level, PBMT has been shown to enhance ATP production, regulate reactive oxygen species, and promote tissue regeneration, thereby supporting mucosal healing and reducing inflammatory responses (11). Compared with standard supportive care alone, PBMT has shown therapeutic potential in mitigating mucosal injury and alleviating associated pain in cancer patients (12).

Systematic reviews published within the last few years report that PBMT is effective in reducing the incidence and severity of OM in patients undergoing chemotherapy, radiotherapy, or combined chemoradiotherapy (5, 10, 13). Meta-analytical evidence further demonstrates that PBMT significantly decreases the severity of oral mucositis and improves clinical outcomes compared with placebo or standard care, with moderate certainty of evidence (14). Furthermore, recent clinical evidence indicates that PBMT use is associated with improvements in patient-reported outcomes such as oral pain and quality of life, and that it may be cost-effective as an adjunct to conventional care in OM management (15, 16). Consequently, international clinical practice guidelines, including those developed by the MASCC/ISOO Mucositis Study Group, recommend PBMT for the prevention and management of OM in selected patient populations (17).

Although numerous systematic reviews and clinical trials have evaluated the effectiveness of PBMT, the existing literature is predominantly focused on clinical outcomes and lacks a comprehensive understanding of the global research landscape. In particular, there is limited insight into how scientific production has evolved over time, which authors and institutions drive the field, and how international collaboration networks shape knowledge development. Furthermore, the absence of structured mapping of thematic trends limits the ability to identify emerging research directions and gaps in the evidence base.

This lack of a comprehensive bibliometric perspective hinders the strategic development of the field, as it restricts the identification of research priorities, collaboration opportunities, and areas requiring methodological standardization. Therefore, a systematic evaluation of publication patterns, citation impact, and research networks is essential to better understand the intellectual structure of PBMT research and to support its more effective integration into evidence-based oncology practice.

Bibliometric analysis provides a quantitative method to map the development of a research field by describing patterns of scientific output, identifying influential contributors, and revealing thematic trends over time. Unlike traditional systematic reviews focused on clinical effectiveness, bibliometrics elucidates the intellectual structure and evolution of research activity within a domain (18).

In this context, the present study aims to address this gap by mapping the global scientific landscape of PBMT research related to chemotherapy- and radiotherapy-induced oral mucositis by evaluating publication trends, key authors and institutions, collaboration networks, and thematic evolution using bibliometric indicators and science-mapping techniques.

## 2 Methods

### 2.1 Study design and reporting guidelines

This study was designed as a bibliometric analysis to evaluate global research trends in photobiomodulation therapy for chemotherapy- and radiotherapy-induced oral mucositis. The study focuses on bibliometric mapping, scientific productivity, citation dynamics, and research collaboration rather than the quantitative synthesis of clinical outcomes.

The reporting of the literature identification and selection process was guided by the Preferred Reporting Items for Systematic Reviews and Meta-Analyses (PRISMA 2020) statement to ensure transparency and reproducibility. However, this study does not represent a systematic review or meta-analysis, as no qualitative or quantitative synthesis of clinical evidence was performed.

### 2.2 Data sources

A comprehensive bibliographic search was conducted in PubMed, Scopus, and Web of Science Core Collection to identify publications related to PBMT for chemotherapy- and radiotherapy-induced oral mucositis. We included publications indexed between 1 January 2016, and 31 December 2025; the search was conducted on 3 January 2026.

### 2.3 Search strategy

The complete search strategies applied in PubMed, Scopus, and Web of Science, including all search terms, Boolean operators, and database-specific filters, are provided in Supplementary Material (Supplementary Tables A.1–A.3).

The literature search was structured around three main conceptual domains: (1) oral mucositis, (2) anticancer treatment modalities (chemotherapy, radiotherapy, or chemoradiotherapy), and (3) photobiomodulation therapy–related terminology. These domains were combined using appropriate Boolean operators (AND, OR).

Representative keywords included “oral mucositis,” “photobiomodulation therapy,” “low-level laser therapy,” “PBMT,” “LLLT,” “chemotherapy,” “radiotherapy,” and “chemoradiotherapy.” Controlled vocabulary terms (e.g., MeSH in PubMed) and free-text terms were used where appropriate.

Search filters were applied to limit results to English-language publications, articles and review articles, and studies published between 2016 and 2025.

### 2.4 PICOS framework

The research question was defined using the PICOS framework.

Population/unit of analysis: peer-reviewed publications on cancer therapy–induced oral mucositis.

Intervention: Photobiomodulation therapy, low-level laser therapy, or related light-based therapeutic modalities.

Comparator: Not applicable, as this study aimed to evaluate research trends rather than clinical effectiveness.

Outcomes: Bibliometric indicators including publication trends, citation analysis, authorship patterns, institutional productivity, international collaboration, and keyword evolution.

### 2.5 Eligibility criteria

The eligibility criteria were defined prior to the screening process to ensure consistency and reproducibility of study selection.

#### 2.5.1 Inclusion criteria

Studies were included if they met the following criteria:

(i) publications focused on photobiomodulation therapy in the context of chemotherapy- and/or radiotherapy-induced oral mucositis;
(ii) articles indexed in PubMed, Scopus, or Web of Science;
(iii) original research articles or review articles;
(iv) publications written in English; and
(v) studies published between 1 January 2016, and 31 December 2025.

#### 2.5.2 Exclusion criteria

Studies were excluded if they met any of the following criteria:

(i) conference abstracts, editorials, letters, and other non-peer-reviewed publications;
(ii) duplicate records;
(iii) animal studies;
(iv) *in vitro* studies; and
(v) studies not addressing PBMT for OM

The eligibility criteria were applied in a two-stage screening process. After removal of duplicate records, titles and abstracts were screened to exclude clearly irrelevant records, including animal studies, *in vitro* studies, and studies not related to photobiomodulation therapy and oral mucositis.

In addition, database-level filters were applied to include only peer-reviewed journal publications, thereby excluding conference abstracts, editorials, letters, and other non-peer-reviewed document types.

Following screening, all remaining records that met the predefined eligibility criteria were included in the final bibliometric dataset.

### 2.6 Data export and merging

Bibliographic metadata, including titles, authors, institutional affiliations, keywords, citation counts, and publication years, were exported in BibTeX and plain text formats and processed in RStudio (version 2024.12.1) using the bibliometrix package. All records were merged and screened prior to analysis. Bibliometric analyses were conducted using the bibliometrix R package (version 4.5.2) and the Biblioshiny (version 5.2.1) web interface. The analyses comprised descriptive performance indicators—such as annual publication trends, citation metrics, and the most influential authors, institutions, and journals—as well as science mapping techniques. Network analyses were applied to explore coauthorship patterns, keyword co-occurrence, and thematic evolution of the research field. Additional network visualizations, including chord diagrams of international collaboration, were generated using the RAWGraphs web-based visualization platform. To ensure accuracy and reproducibility, all bibliometric analyses were independently verified by two authors (MA and AT).

## 3 Results

### 3.1 Dataset characteristics

**Figure 1:**
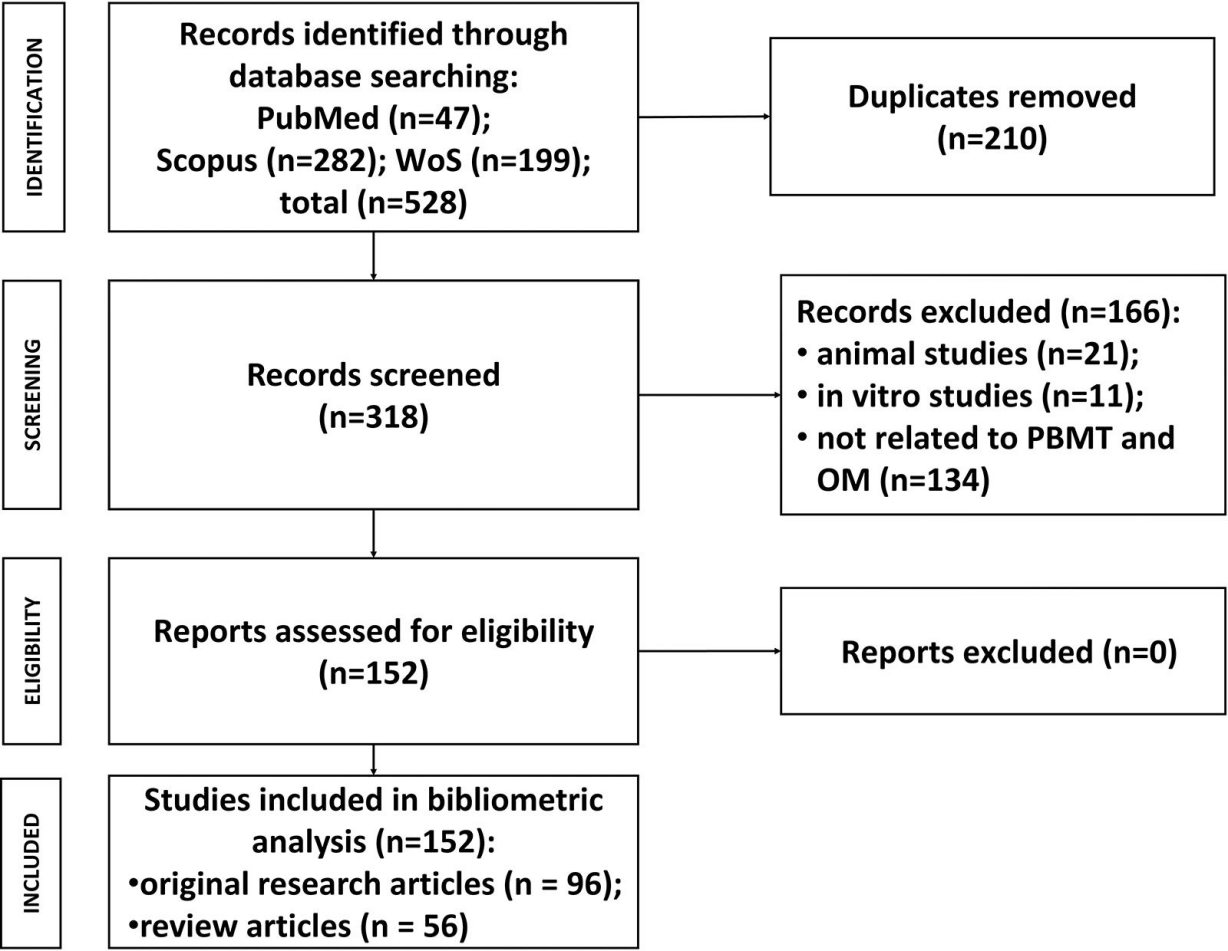
A flow diagram of the literature search and study selection process. The diagram shows identification of records from PubMed, Scopus, and Web of Science, removal of duplicates, screening process, and final inclusion of studies in the bibliometric analysis.

The initial database search identified 47 records from PubMed, 282 records from Scopus, and 199 records from Web of Science, yielding a total of 528 records. After removal of 210 duplicate records, 318 unique records remained for title and abstract screening. During the screening process, 166 records were excluded, including animal studies (*n* = 21), *in vitro* studies (*n* = 11), and studies not meeting the predefined eligibility criteria (*n* = 134). These included studies not related to photobiomodulation therapy, studies not addressing chemotherapy- or radiotherapy-induced OM, and publications outside the scope of the bibliometric analysis. Following screening, 152 studies met the eligibility criteria and were included in the final bibliometric analysis (Figure 1).

### 3.2 Annual scientific production

**Figure 2:**
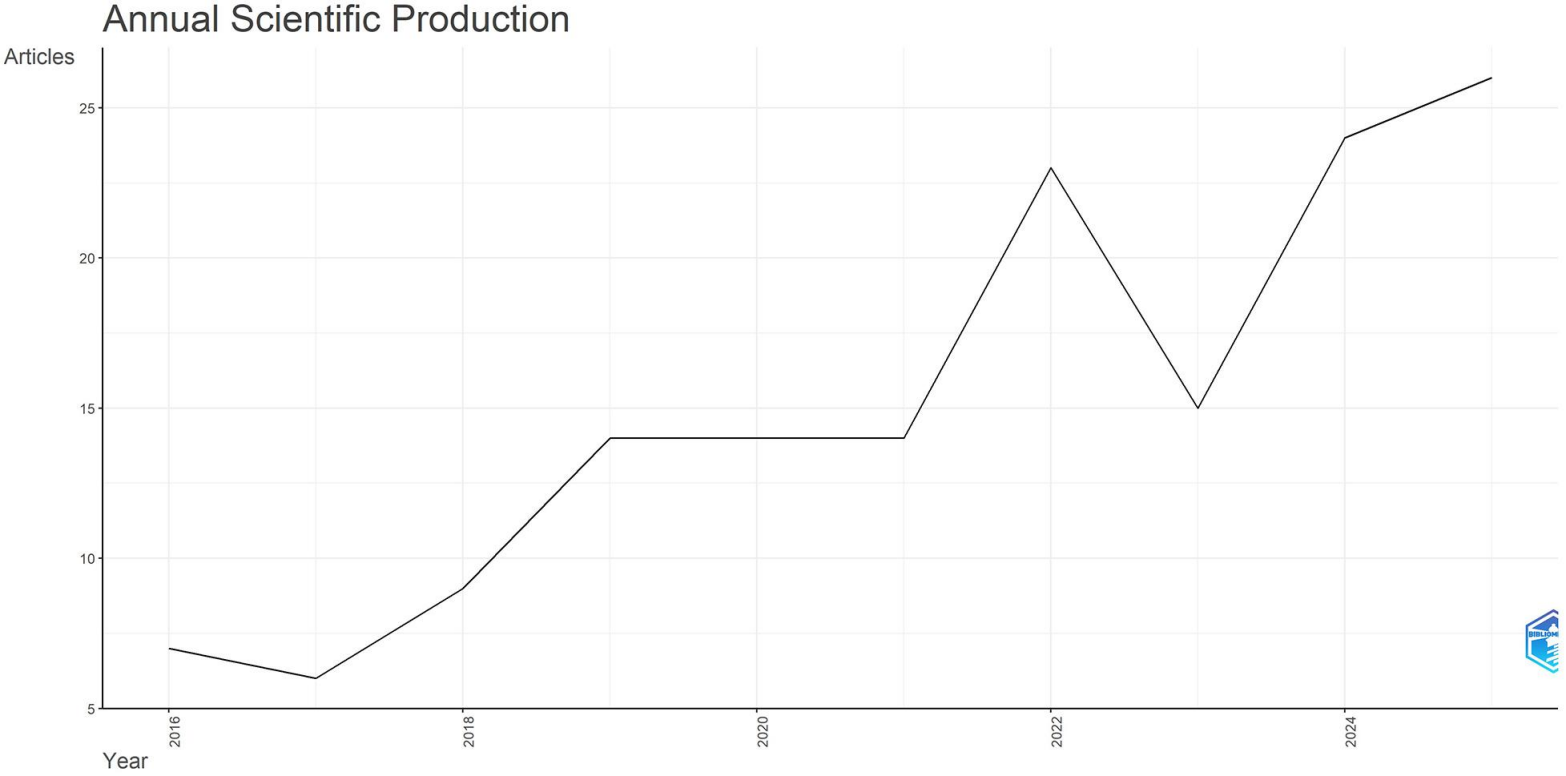
Annual scientific production of publications on photobiomodulation therapy for chemotherapy- and radiotherapy-induced oral mucositis from 2016 to 2025. The graph demonstrates a progressive increase in research output over time.

Annual publication output demonstrated a clear upward trend over the study period (Figure 2). After a relatively low activity in 2016–2017, production increased steadily from 2018, likely reflecting growing clinical interest and the publication of international guidelines (17, 19), with a plateau during 2019–2021. A notable peak occurred in 2022, potentially associated with increased dissemination of clinical evidence and guideline updates, followed by a temporary decline in 2023. Subsequently, publication output rose sharply, reaching its highest level in 2025, which may indicate renewed research momentum and expanding clinical adoption of PBMT.

### 3.3 Sources (journals)

#### 3.3.1 Most relevant sources

**Figure 3:**
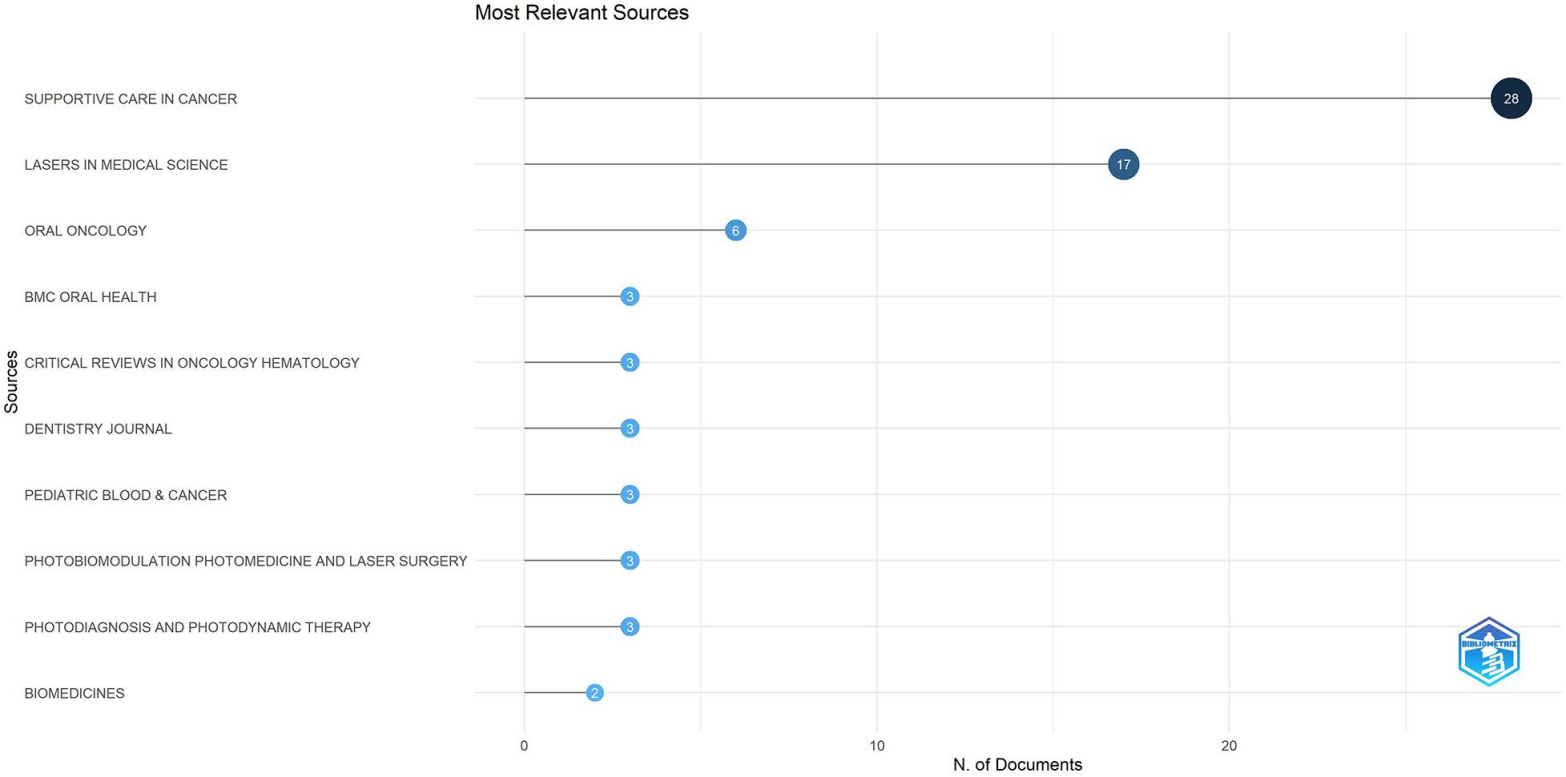
Most relevant journals publishing research on photobiomodulation therapy for oral mucositis. Bubble size represents the number of publications per journal.

Research output was concentrated in a limited number of specialized journals (Figure 3). *Supportive Care in Cancer* was the leading source (*n* = 28), followed by *Lasers in Medical Science* (*n* = 17). Other journals contributed substantially fewer publications, indicating a centralized publication structure within a small group of core outlets, likely reflecting the interdisciplinary nature of PBMT research spanning oncology and photomedicine.

#### 3.3.2 Sources’ production over time

**Figure 4:**
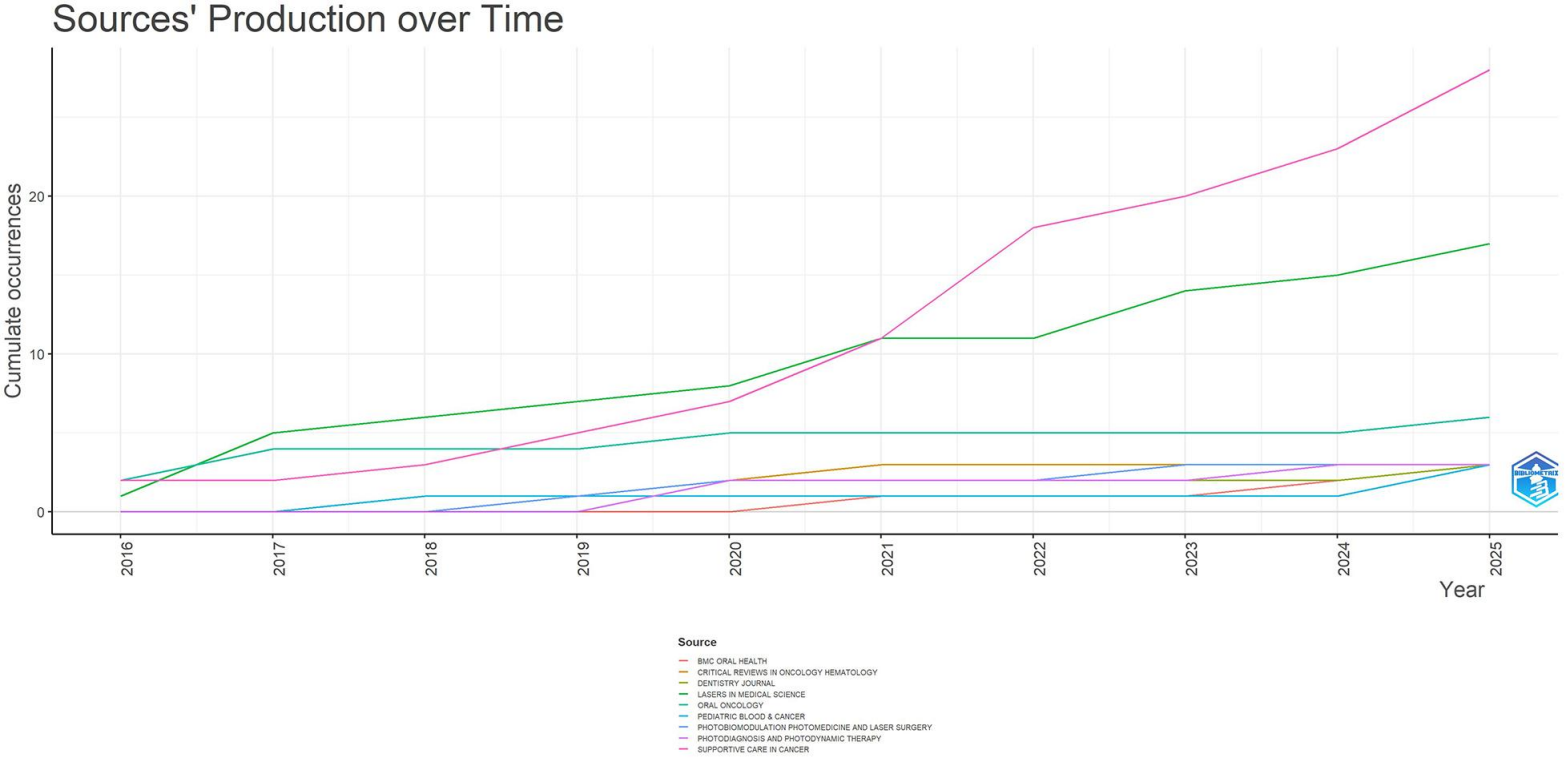
Sources’ production over time showing cumulative publication output of leading journals. The trends demonstrate sustained contributions from key journals and highlight the progressive consolidation of PBMT research within established publication platforms.

Publication output across leading journals increased steadily after 2018 (Figure 4). *Supportive Care in Cancer* showed the most pronounced growth, particularly after 2020, likely due to its central role in publishing supportive oncology research and clinical guidelines (20). *Lasers in Medical Science* demonstrated consistent long-term productivity, reflecting sustained interest in laser-based therapeutic applications. Other journals exhibited lower but gradually increasing output, suggesting a broader dissemination of PBMT research across related disciplines.

### 3.4 Publications

**Figure 5:**
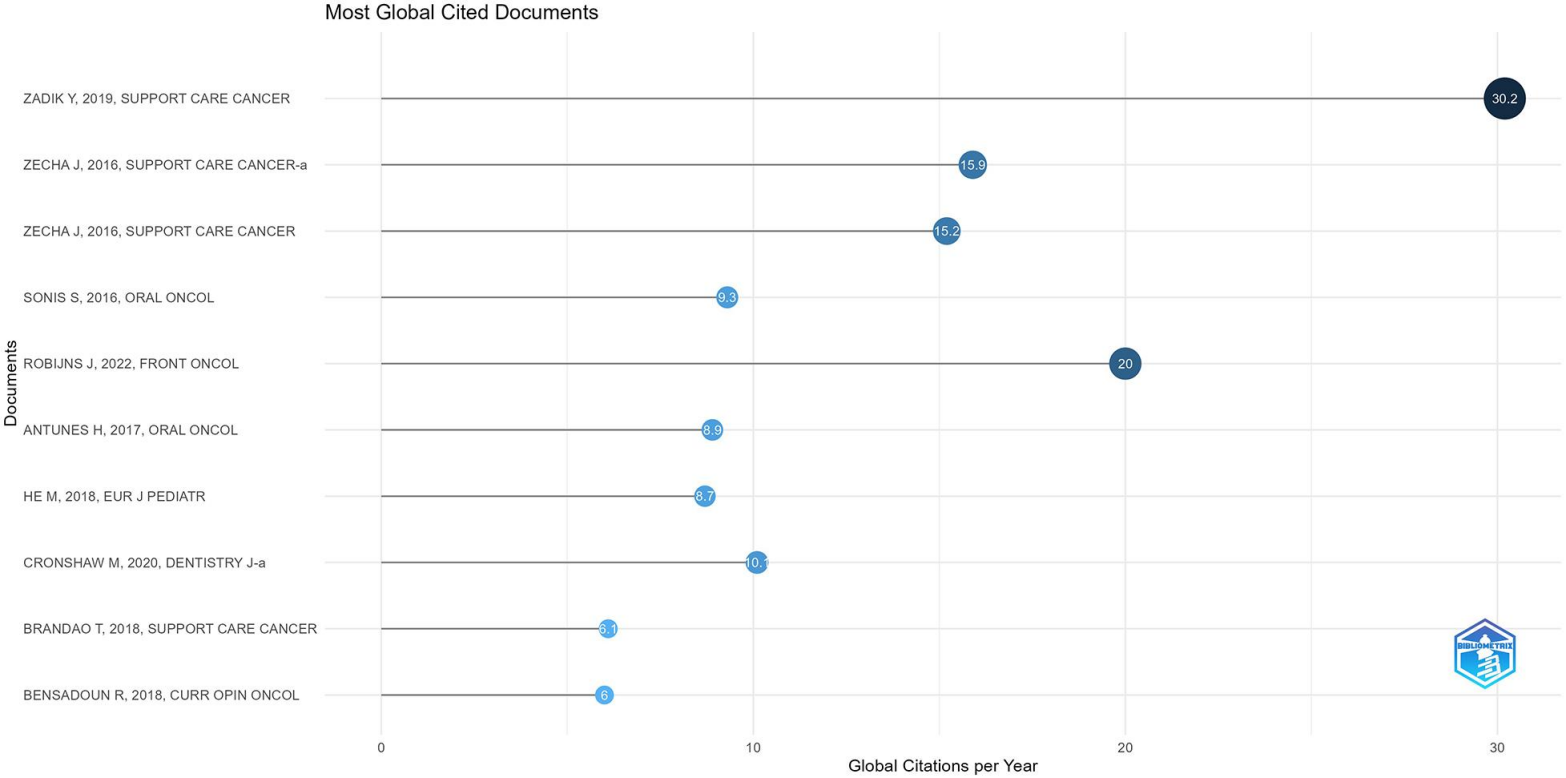
Time-normalized citation impact of the top 10 most influential publications on photobiomodulation therapy for chemotherapy- and radiotherapy-induced oral mucositis.

Table 1 presents the top 10 most influential publications on photobiomodulation therapy for chemotherapy- and radiotherapy-induced oral mucositis, including both total citations and time-normalized citation metrics (citations per year) to reduce bias associated with publication age. Figure 5 illustrates the time-normalized citation impact of these publications, allowing comparison across different publication years.

**Table 1:** Top 10 publications ranked by total citations and citations per year in photobiomodulation therapy for oral mucositis.

First author, year	Title	Source	Total citations	Citations per year
Zadik, 2019 (17)	Systematic review of photobiomodulation for the management of oral mucositis in cancer patients and clinical practice guidelines	*Support Care Cancer*	242	30.25
Zecha, 2016 (40)	Low-level laser therapy/photobiomodulation in the management of side effects of chemoradiation therapy in head and neck cancer: part 1: mechanisms of action, dosimetric, and safety considerations	*Support Care Cancer*	175	15.91
Zecha, 2016 (32)	Low-level laser therapy/photobiomodulation in the management of side effects of chemoradiation therapy in head and neck cancer: part 2: proposed applications and treatment protocols	*Support Care Cancer*	167	15.18
Sonis, 2016 (48)	Could the biological robustness of low-level laser therapy (photobiomodulation) impact its use in the management of mucositis in head and neck cancer patients	*Oral Oncol*	102	9.27
Robijns, 2022 (21)	Photobiomodulation therapy in the management of cancer therapy–induced side effects: WALT position paper 2022	*Front Oncol*	100	20.00
Antunes, 2017 (49)	Long-term survival of a randomized phase III trial of head and neck cancer patients receiving concurrent chemoradiation therapy with or without low-level laser therapy (LLLT) to prevent oral mucositis	*Oral Oncol*	89	8.90
He, 2018 (50)	A systematic review and meta-analysis of the effect of LLLT on chemotherapy-induced oral mucositis in pediatric and young patients	*Eur J Pediatr*	78	8.67
Cronshaw, 2020 (51)	Photobiomodulation and Oral Mucositis: A Systematic Review	*Dentistry J*	71	10.14
Brandao, 2018 (34)	Locally advanced oral squamous cell carcinoma patients treated with photobiomodulation for prevention of oral mucositis: retrospective outcomes and safety analyses	*Support Care Cancer*	55	6.11
Bensadoun, 2018 (33)	Photobiomodulation or low-level laser therapy in the management of cancer therapy–induced mucositis, dermatitis, and lymphedema	*Curr Opin Oncol*	54	6.00

The most highly cited article was the systematic review and clinical practice guidelines by Zadik et al. (17), with 242 total citations and the highest citation rate (30.25 citations per year). Notably, more recent publications such as Robijns et al. (21) demonstrated high citation rates despite lower total citation counts, indicating rapidly growing scientific influence within the field.

The analysis of photobiomodulation parameters showed that the majority of studies employed wavelengths within the red and near-infrared spectrum, typically ranging from approximately 630–980 nm. The most frequently reported wavelengths were centered around 660 and 810–830 nm, either used individually or in combination (22–26).

Laser-based photobiomodulation, particularly diode lasers, was the most commonly used modality across the included studies. LED-based systems were reported less frequently (21, 27–29). With regard to emission mode, continuous wave irradiation was more commonly reported than pulsed delivery. However, reporting of emission parameters was inconsistent, and only a limited number of studies explicitly described pulsed protocols (25, 30–32). Considerable variability was observed in treatment parameters such as energy density, treatment frequency, and protocol design.

### 3.5 Authors

#### 3.5.1 Most relevant authors

**Figure 6:**
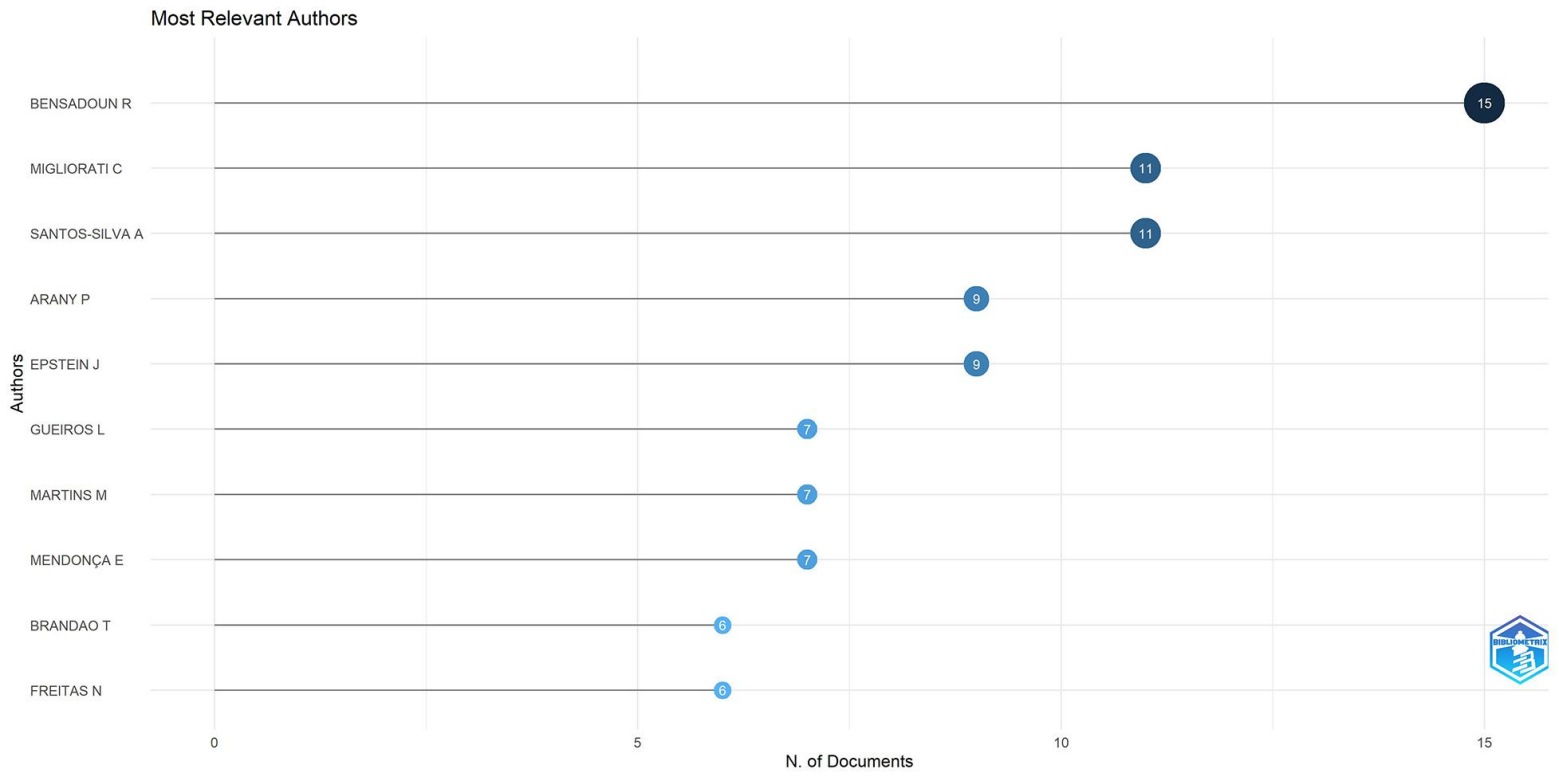
Most productive authors contributing to PBMT research. Bubble size indicates the number of publications per author. The results reveal a limited group of highly active researchers driving scientific output in this domain.

Research productivity in PBMT-related oral mucositis research was concentrated among a limited number of authors (Figure 6). Bensadoun was the most prolific contributor (*n* = 15), followed by Santos-Silva (*n* = 13), and Arany and Migliorati (*n* = 12 each). Other active contributors included Epstein (*n* = 9), Gueiros, Martins, and Mendonça (*n* = 7 each), as well as Brandão and Freitas (*n* = 6 each). Overall, the findings indicate a relatively concentrated authorship structure within the field.

#### 3.5.2 Authors’ production over time

**Figure 7:**
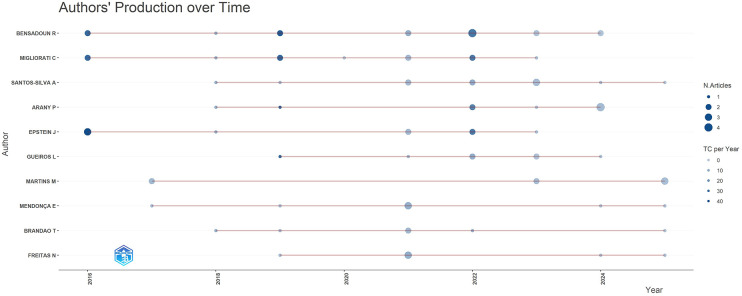
Authors’ production over time showing annual publication activity and citation impact of leading authors. The figure illustrates sustained research engagement by key contributors and increasing citation impact in recent years, reflecting their ongoing influence in the field.

The temporal evolution of authors’ publication activity is presented in Figure 7. Several key contributors, including Bensadoun (33), Migliorati (27), Santos-Silva (34), and Arany (35), demonstrated sustained research activity over multiple years. Early contributions were observed for Bensadoun, Migliorati, and Epstein, while increased publication activity and citation impact were evident after 2018, particularly during 2021–2022. These trends reflect the growing influence and continued engagement of leading authors in the development of PBMT research.

### 3.6 Affiliations

#### 3.6.1 Most relevant affiliations

**Figure 8:**
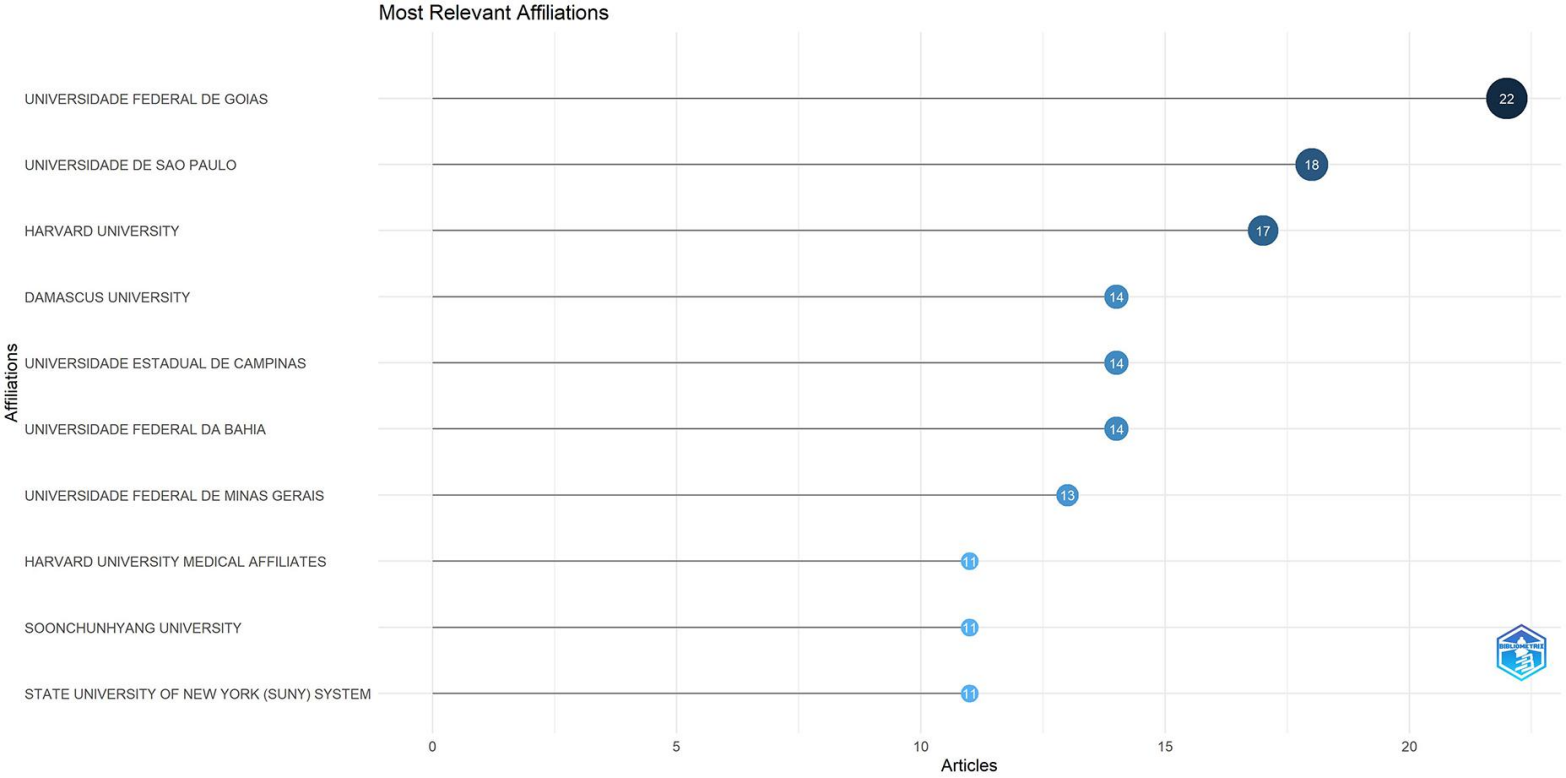
Most relevant institutional affiliations contributing to PBMT research. The distribution highlights the dominance of several key institutions, indicating institutional clustering of expertise and research activity.

Institutional productivity was concentrated among a limited number of leading centers (Figure 8). The Universidade Federal de Goiás (36) was the most productive institution (*n* = 22), followed by the Universidade de São Paulo (37) (*n* = 18) and Harvard University (32) (*n* = 17), indicating strong contributions from both Latin American and North American institutions. Other active contributors included Damascus University, Universidade Estadual de Campinas, and Universidade Federal da Bahia (*n* = 14 each), as well as Universidade Federal de Minas Gerais (*n* = 13). Overall, the findings demonstrate a geographically diverse but relatively concentrated institutional structure.

#### 3.6.2 Affiliations’ production over time

**Figure 9:**
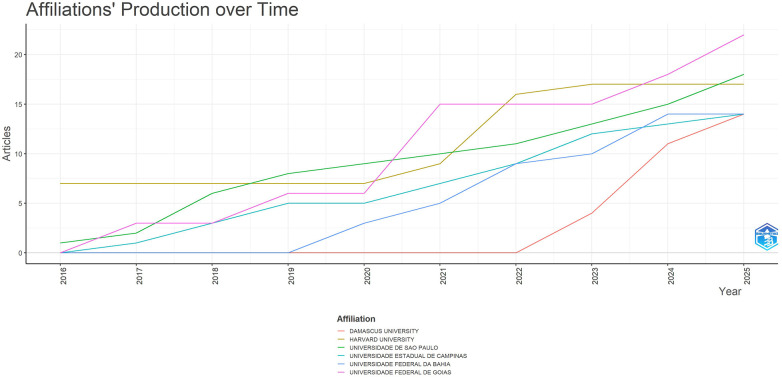
Institutional scientific production over time showing cumulative output of leading affiliations. The trends suggest stable and long-term institutional involvement, with some centers demonstrating consistent growth in research productivity.

The temporal evolution of institutional productivity is shown in Figure 9. Overall, publication output increased across leading institutions, particularly after 2019. The Universidade Federal de Goiás demonstrated the most pronounced growth, reaching the highest cumulative output by 2025. Universidade de São Paulo and Universidade Estadual de Campinas showed steady and sustained contributions, while Harvard University exhibited consistent early activity with increased output after 2021. In contrast, Damascus University showed later but rapid growth, with publications emerging after 2022. These trends highlight both established and emerging institutional contributors and reflect the expanding global engagement in PBMT research.

### 3.7 Countries

#### 3.7.1 Most cited countries

**Figure 10:**
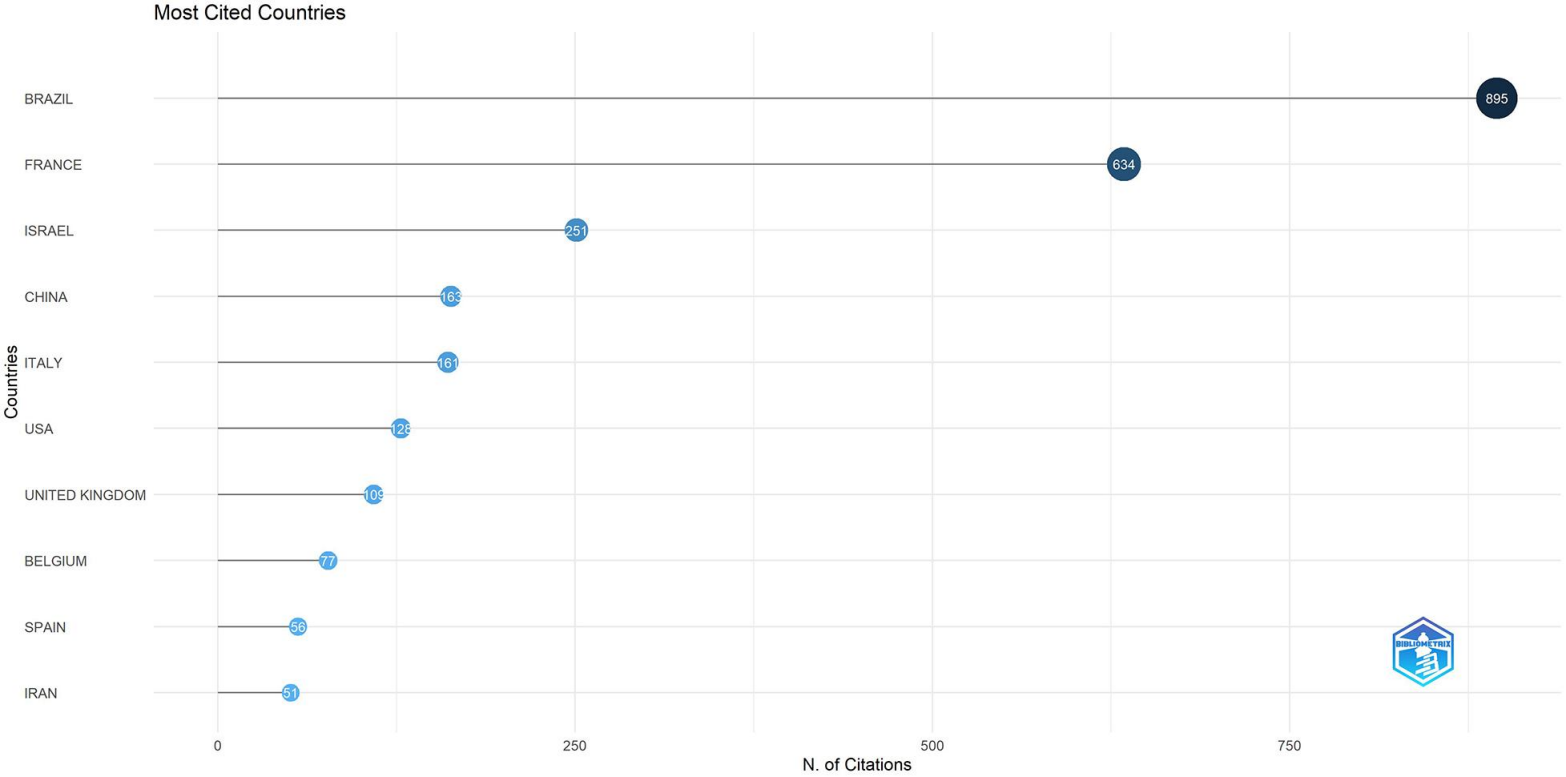
Most cited countries contributing to PBMT research. Bubble size represents total citation count. The results indicate that a small number of countries account for a disproportionately high share of global scientific impact.

The geographical distribution of citations is presented in Figure 10. Brazil demonstrated the highest citation impact (*n*=895), followed by France (*n*=634), indicating their leading role in PBMT research. Other countries such as Israel, China, and Italy contributed moderate citation outputs, while the United States and the United Kingdom also showed notable impact. Additional contributions from Belgium, Spain, and Iran reflect broader international engagement, although with comparatively low citation levels.

#### 3.7.2 Corresponding author's countries

**Figure 11:**
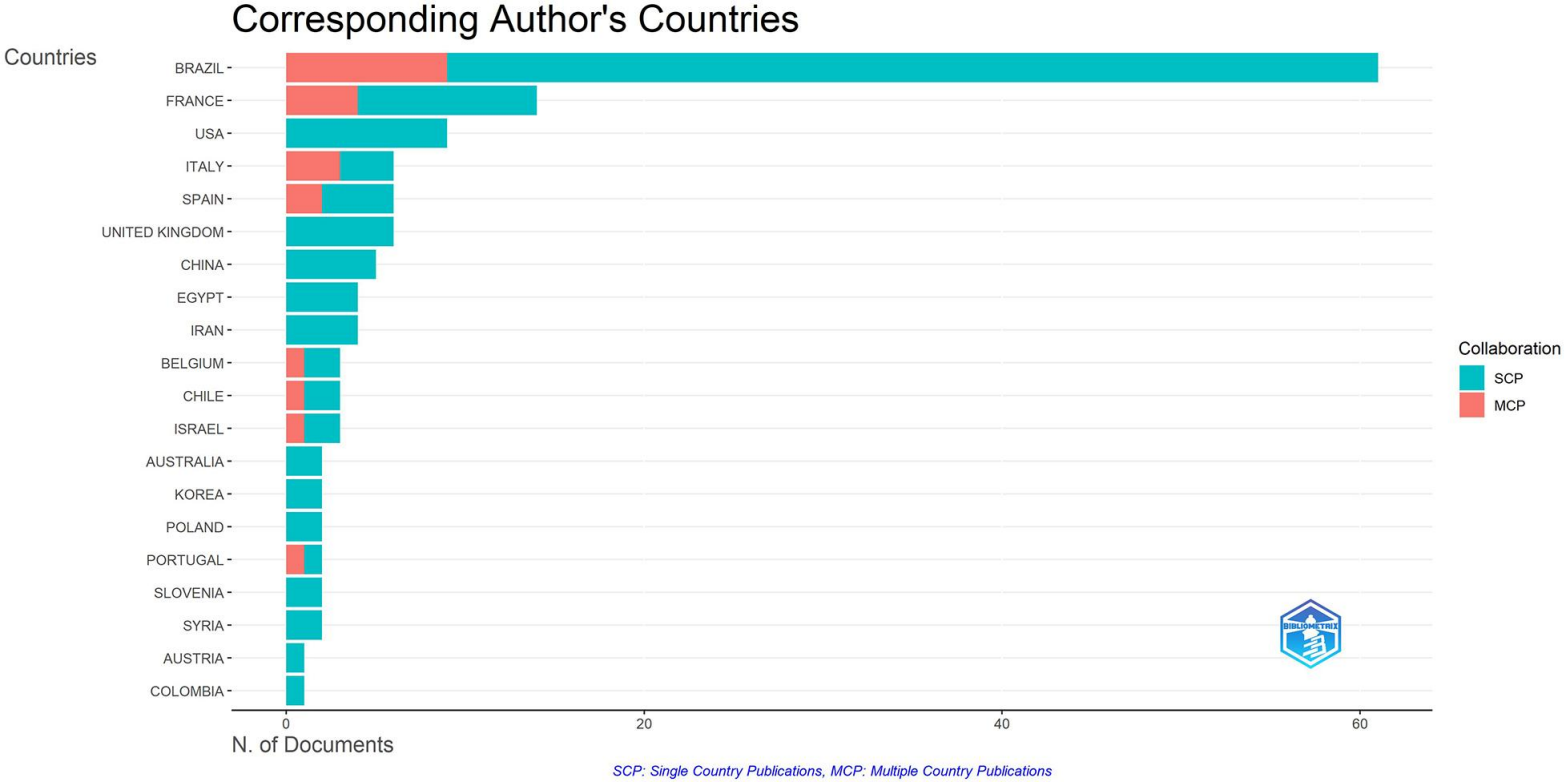
Corresponding authors’ countries showing single-country publications (SCPs) and multiple-country publications (MCPs). SCPs indicate studies conducted within one country, while MCPs reflect international collaboration.

The distribution of corresponding authors is shown in Figure 11, distinguishing between single-country and multicountry publications. Brazil was the most productive country, with a predominance of single-country publications, indicating strong national research capacity (36, 38). France ranked second, demonstrating a balanced contribution of domestic and international collaborations (35, 39). The United States, Italy, Spain, and the United Kingdom also showed notable authorship activity, with varying levels of international collaboration. Other countries contributed fewer publications, highlighting a globally distributed but uneven pattern of research leadership.

#### 3.7.3 Countries’ productivity over time

**Figure 12:**
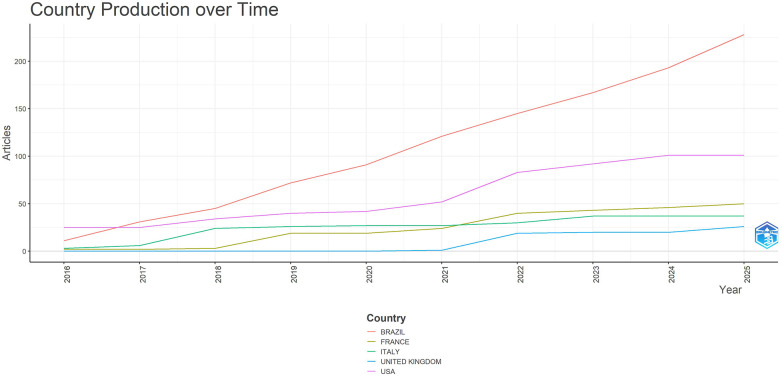
Country scientific production over time showing annual publication trends of leading countries. The trends reveal sustained growth in output from key countries, with emerging contributions from additional regions.

The temporal evolution of publication output by country is shown in Figure 12. Overall, leading countries demonstrated a consistent increase in research activity over time. Brazil showed the most pronounced and sustained growth, reaching the highest cumulative output by 2025. The United States also exhibited substantial growth, particularly after 2021. In contrast, France and Italy showed steady but moderate increases, while the United Kingdom demonstrated a later but gradually increasing contribution. These trends reflect the expanding global engagement in PBMT research.

#### 3.7.4 International collaboration network

**Figure 13:**
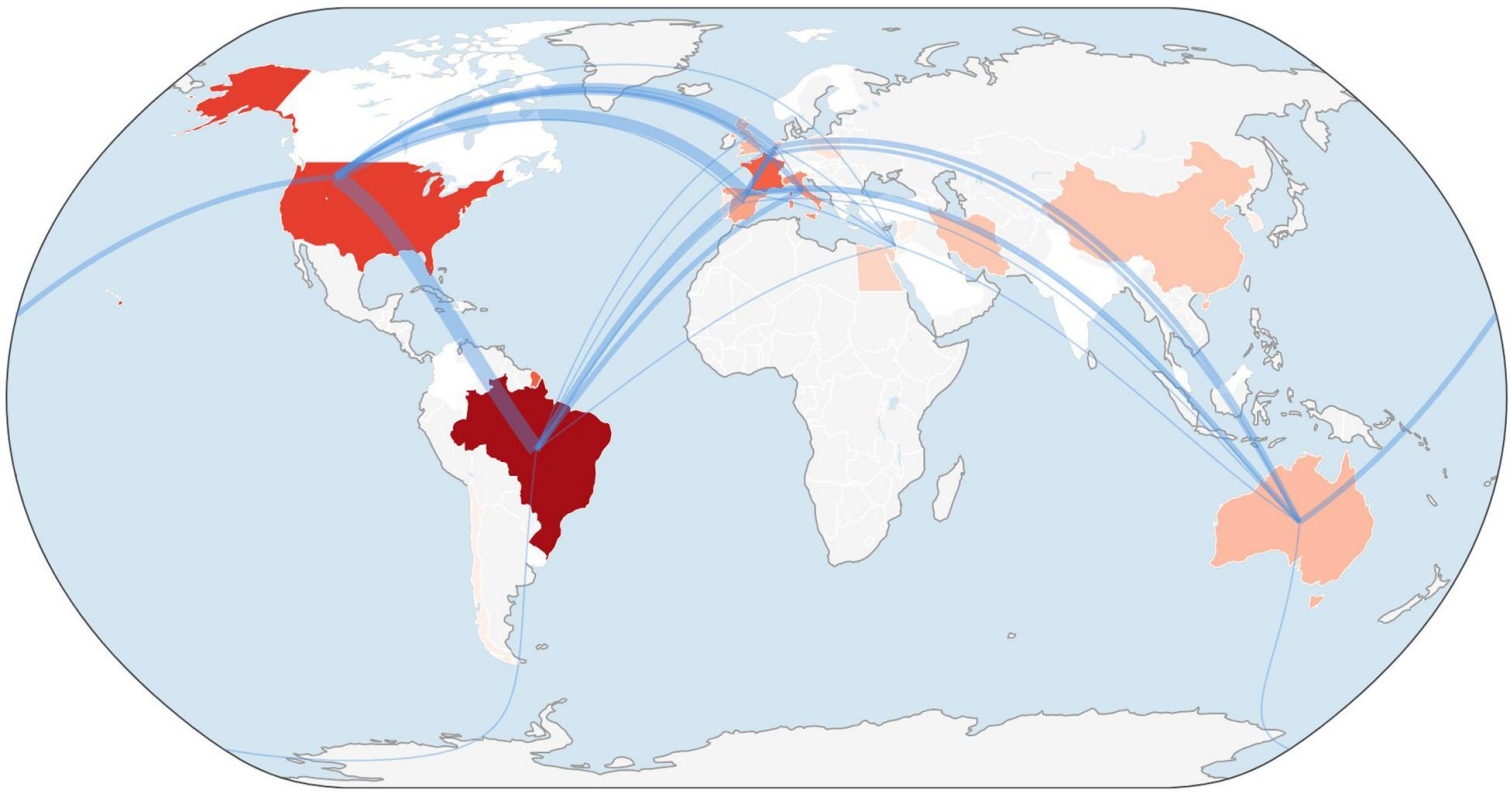
A global collaboration map illustrating international research partnerships. Dense connections between Europe, North America, and Brazil highlight strong transcontinental collaboration, while emerging links from Asia indicate expanding global participation.

**Figure 14:**
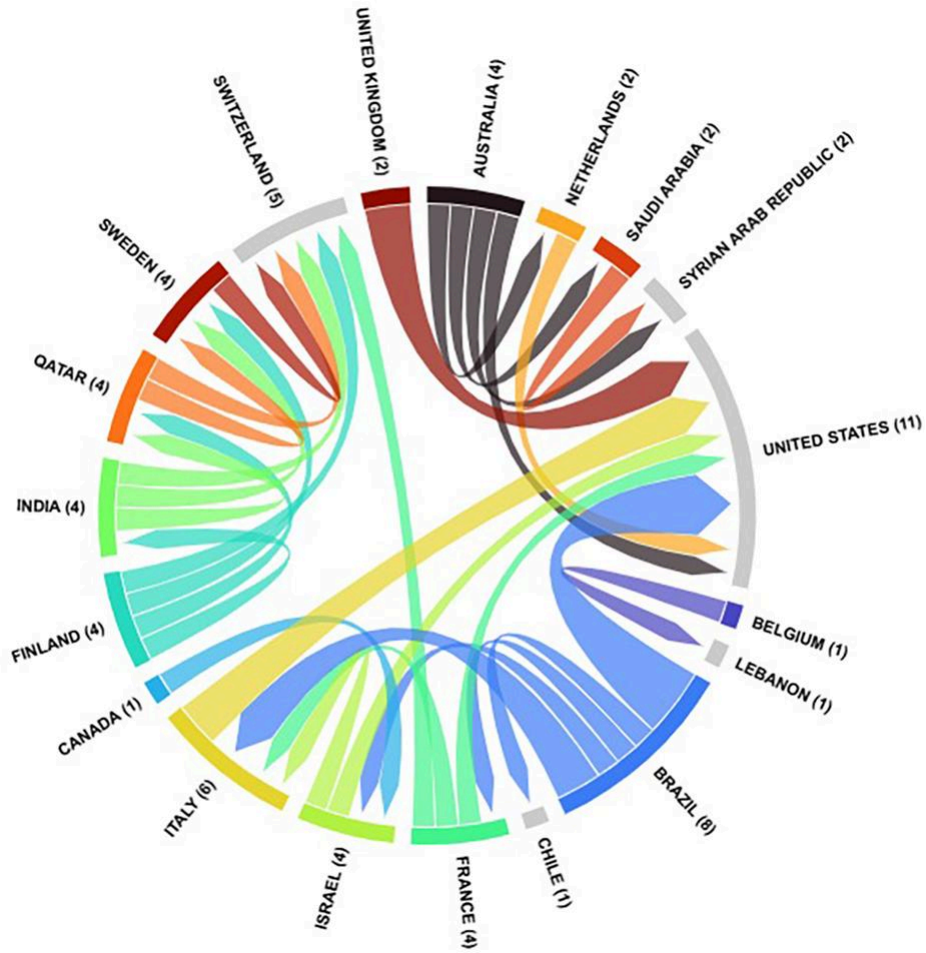
An international collaboration network showing coauthorship between countries. Brazil and the United States act as central hubs within a highly interconnected global research network.

International collaboration patterns are illustrated in Figures 13, 14. Brazil and the United States emerged as leading contributors, combining high publication output with extensive international collaboration and functioning as central hubs within the global network (34). Strong partnerships were observed across Europe, as well as between Europe, North America, and Australia (19, 21). Brazil demonstrated particularly extensive connections with both North America and European countries, highlighting its pivotal role in PBMT research (40).

The overall topology suggests a core–periphery structure, where a group of highly collaborative countries drives research activity, while other regions, including parts of Asia and the Middle East, show emerging but less integrated participation.

### 3.8 Trend topics and words’ frequency over time

**Figure 15:**
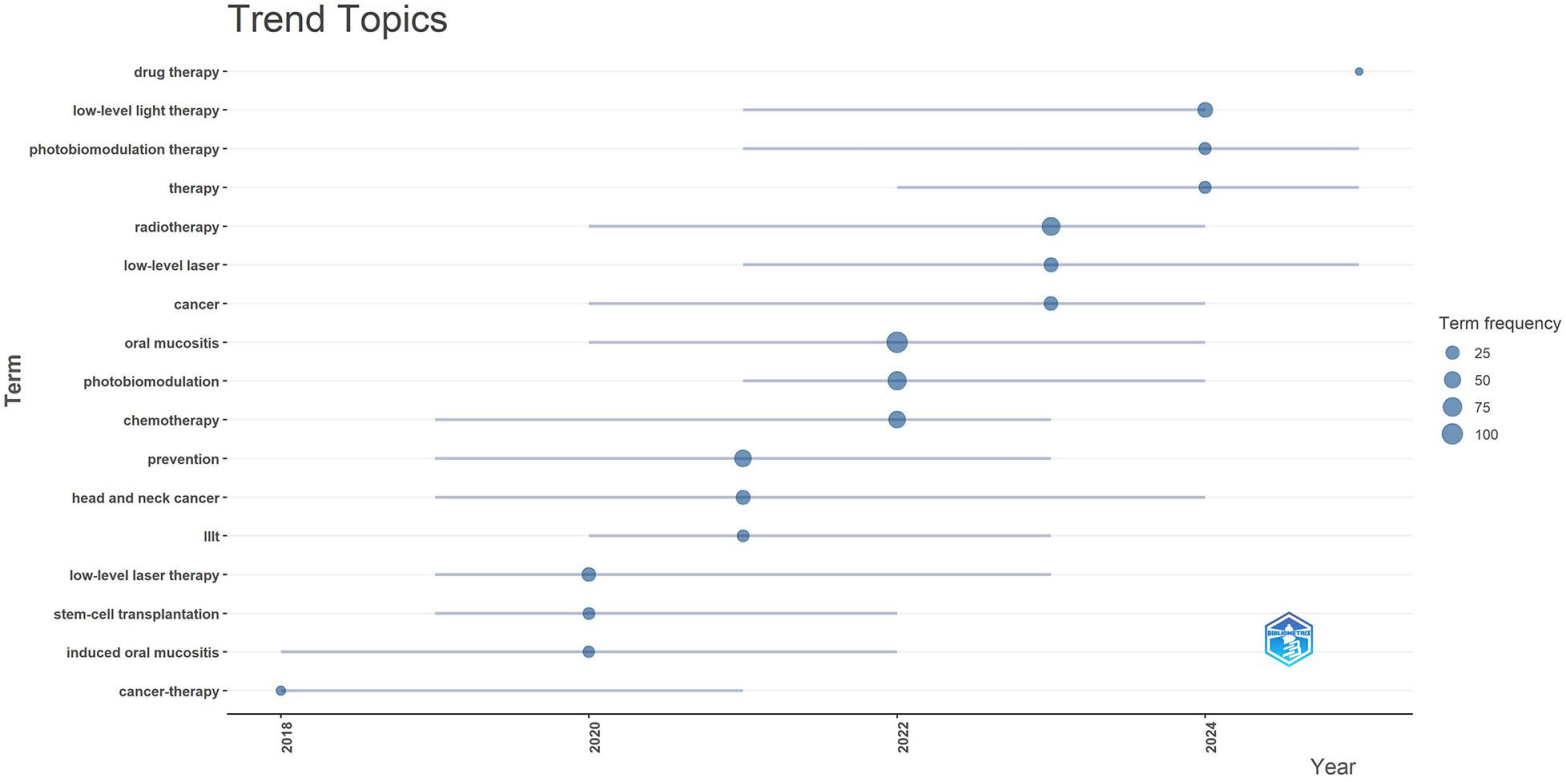
Trend topics showing a temporal evolution of research keywords in PBMT. The shift in keywords over time reflects the transition from foundational studies toward more clinically oriented and outcome-focused research.

The temporal evolution of research topics is shown in Figure 15. Early studies (2016–2018) were characterized by broader terms such as “low-level laser therapy” and general cancer-related concepts. From 2019 onward, research focus shifted toward more clinically oriented topics, including chemotherapy, radiotherapy, and prevention. In recent years (2022–2025), terms such as “photobiomodulation therapy” and “oral mucositis” became dominant, reflecting the consolidation of PBMT as a key therapeutic approach. The emergence of drug-related terms further suggests increasing interest in multimodal supportive care strategies. Overall, these trends indicate progressive conceptual maturation and growing clinical relevance of PBMT research.

## 4 Discussion

This bibliometric analysis provides a comprehensive overview of research trends, thematic evolution, and collaborative patterns in PBMT for chemotherapy- and radiotherapy-induced oral mucositis over the period from 2016 to 2025. The findings demonstrate a clear and sustained increase in annual scientific production, particularly from 2018 onward, with the highest publication output observed in 2025. This trend may reflect the increasing integration of PBMT into evidence-based supportive oncology frameworks, particularly following the publication of international clinical guidelines, rather than merely a general increase in research activity.

However, the increasing volume of publications should not be interpreted as a direct indicator of robust clinical evidence. Recent meta-analyses and systematic reviews (5, 11, 13, 14) consistently demonstrate that PBMT significantly reduces the severity, incidence, and pain associated with oral mucositis; for example, pooled analyses of randomized controlled trials report a statistically significant reduction in mucositis severity compared with control interventions (22). Nevertheless, these findings should be interpreted with caution, as many included randomized controlled trials are characterized by small sample sizes, heterogeneity in study design, and inconsistent outcome measures, which may reduce the reliability and generalizability of pooled estimates. This discrepancy between growing publication volume and persistent methodological limitations highlights a critical gap between research productivity and the robustness of clinical evidence.

The findings indicate that photobiomodulation is most commonly performed using red and near-infrared wavelengths, particularly around 660 and 810–830 nm. These wavelengths are known to interact with mitochondrial chromophores, particularly cytochrome c oxidase, leading to increased ATP production and modulation of inflammatory pathways (41). Diode laser systems are used more frequently than LED devices, reflecting their broader clinical application and ease of use in dental practice. In contrast, LED-based approaches appear to be less commonly applied and are typically used as adjunctive methods. These mechanistic effects provide a biological rationale for PBMT; however, the clinical translation of these mechanisms depends on precise treatment parameters, which may partly explain differences in reported outcomes.

From a clinical perspective, photobiomodulation therapy is primarily applied as a preventive or early-stage intervention during chemotherapy and radiotherapy. It is typically delivered intraorally at specific mucosal sites using low energy densities, with repeated applications throughout the treatment course, often several times per week depending on the oncology regimen. Preventive protocols are generally initiated before or at the start of cancer therapy, whereas therapeutic applications are implemented at the first signs of mucosal inflammation to reduce the onset and severity of oral mucositis.

Comparative bibliometric studies examining OM research beyond the PBMT context also report parallel growth patterns, with analyses highlighting the increasing role of journals such as Supportive Care in Cancer as central platforms for disseminating work in this field and revealing expanding research activity and thematic clusters within the broader OM literature (42). Similar findings have been reported in large-scale bibliometric analyses of cancer therapy–induced mucosal injury, which demonstrates the growing importance of mechanism-based and supportive care–oriented research directions (43, 44).

Within the PBMT-specific dataset analyzed in the present study, original research articles constituted the majority of publications, indicating a transition from exploratory feasibility studies toward more robust clinical investigations. However, the predominance of systematic reviews and guideline-based publications among the most highly cited articles suggests that citation impact in this field is driven largely by secondary evidence synthesis rather than primary large-scale randomized clinical trials, highlighting a potential imbalance in the evidence hierarchy.

Another salient finding was the concentration of high-impact publications within a relatively small number of specialized journals. In the present analysis, *Supportive Care in Cancer* and *Lasers in Medical Science* emerged as the most influential and productive journals, reinforcing their role as core outlets for OM and PBMT research.

The geographic distribution of PBMT research revealed a pronounced concentration in Brazil and other major Western countries. Brazil, in particular, demonstrated a high volume of publications and citations, corroborating earlier bibliometric assessments that documented the growing prominence of Brazilian research groups in laser and photobiomodulation studies for OM (45, 46). While this reflects strong regional research capacity, it may also introduce geographic bias and limit the external validity and global applicability of findings, particularly in low- and middle-income settings.

A keyword analysis revealed a clear thematic evolution over the study period. Early research was dominated by general terms such as “low-level laser therapy,” whereas more recent publications increasingly employed specific clinical and mechanistic descriptors, including “photobiomodulation therapy,” “prevention,” and “chemotherapy-induced mucositis.” This semantic shift reflects both conceptual maturation and increasing standardization of terminology, although persistent variability in reporting practices indicates that further harmonization is required.

The present study leveraged robust bibliometric methodologies and multiple databases to provide a comprehensive overview of PBMT research in OM. Nevertheless, several limitations should be acknowledged. Some reviews that addressed oral mucositis within a broader spectrum of cancer therapy–related toxicities, such as radiation dermatitis (47) and lymphedema (33), were retained because they placed substantial emphasis on PBMT or low-level laser therapy in the context of OM.

In addition to database and citation bias, selection bias cannot be excluded, as the search strategy and inclusion criteria may have influenced study representation. Furthermore, language bias may have led to an underrepresentation of non-English publications.

Although the present analysis does not directly assess clinical efficacy, it situates PBMT research within the broader framework of supportive oncology care, where accumulating clinical trials and systematic reviews suggest that PBMT improves patient-reported outcomes (5, 11, 13, 14).

Future research should prioritize large-scale, multicenter randomized controlled trials, rigorous standardization of PBMT protocols, and improved reporting transparency. In addition, expanding international collaboration and increasing representation from underreported regions will be critical to enhance the external validity, generalizability, and clinical translation of PBMT research.

## 5 Conclusions

This bibliometric analysis demonstrates a sustained and growing global research interest in photobiomodulation therapy for chemotherapy- and radiotherapy-induced oral mucositis, reflecting its increasing integration into supportive oncology practice. The field is characterized by a rising volume of publications, active international collaboration, and a progressive shift toward clinically oriented and mechanism-based research.

Despite accumulating evidence supporting the beneficial effects of PBMT in reducing the severity and burden of oral mucositis, the overall strength of evidence remains limited by methodological heterogeneity and variability in treatment parameters. This highlights a critical gap between research productivity and the robustness of clinical evidence.

Overall, while PBMT represents a promising and increasingly adopted therapeutic approach, further high-quality, multicenter randomized controlled trials and standardized treatment protocols are essential to ensure reproducibility, improve clinical applicability, and support its consistent implementation in oncology care.

## Data Availability

Publicly available datasets were analyzed in this study. These datasets can be found here: The bibliometric dataset analyzed during this study is included in the Supplementary Material. The dataset was derived from publicly available sources, including Scopus, Web of Science Core Collection, and PubMed.
